# Caution Regarding Self-reported Tramadol Dependence

**DOI:** 10.5811/cpcem.2589

**Published:** 2024-01-25

**Authors:** James Dunford, Aaron Schneir

**Affiliations:** * University of California San Diego, School of Medicine, San Diego, California; † McAlister Institute for Treatment and Education, El Cajon, California; ‡ University of California San Diego Health System, Department of Emergency Medicine, Division of Medical Toxicology, San Diego, California

We are writing in regard to the following article in the current issue of CAL/ACEP’s *Lifeline* Volume 4, 2022–23, which was first published in *Clinical Practice and Cases in Emergency Medicine* in 2022.^1^ In the report, the authors describe an opioid-dependent patient who was initially prescribed tramadol by her primary care physician but who ultimately began traveling to Mexico to purchase escalating doses of tramadol. The authors appropriately highlight the potential nuances of managing tramadol withdrawal versus that from pure opioid agonists. It is certainly possible that the patient was exclusively ingesting tramadol and that buprenorphine was successful in managing the withdrawal from it.

However, we feel obligated to point out a major limitation: that at least as described in the report, there was not testing of the tramadol product she obtained from Mexico nor of the patient to confirm the presence of tramadol and exclude the presence of other opioids such as fentanyl. There is increasing recognition that counterfeit tablets sold ostensibly as controlled substances in Northern Mexican pharmacies may in fact contain illicit drugs such as fentanyl and heroin.^2^ Before this case report continues to be cited as evidence of the successful management of tramadol dependence with buprenorphine, we feel this limitation should be recognized.

Thank you.
